# Impact of glucose metabolism on myocardial fibrosis and inflammation in hypertrophic cardiomyopathy: a cardiac MR study

**DOI:** 10.3389/fendo.2025.1653927

**Published:** 2025-08-26

**Authors:** Xin Peng, Huaibi Huo, Zhengkai Zhao, Qiuyi Cai, Jiangyu Tian, Dandan Yang, Yao Song, Yuheng Huang, Zhuoan Li, Jin Gao

**Affiliations:** ^1^ Department of Radiology, The Third People’s Hospital of Chengdu, Chengdu, China; ^2^ Department of Radiology, The First Hospital of China Medical University, Shenyang, China; ^3^ Department of Radiology, The Fourth Affiliated Hospital of Liaoning University of Traditional Chinese Medicine, Shenyang, China; ^4^ Department of Radiology and Imaging Sciences, Indiana University School of Medicine, Indianapolis, IN, United States; ^5^ Weldon school of Biomedical Engineering, Purdue University, West Lafayette, IN, United States

**Keywords:** hypertrophic cardiomyopathy, HbA1c, cardiac magnetic resonance, myocardial fibrosis, subclinical myocardial inflammation

## Abstract

Diabetes mellitus increases the risk of adverse cardiovascular outcomes in hypertrophic cardiomyopathy (HCM) patients. This retrospective study aimed to evaluate myocardial microstructural alterations, particularly fibrosis and subclinical inflammation, in HCM patients across glycemic statuses using multiparametric cardiac magnetic resonance (CMR). Additionally, it explored the correlation between myocardial fibrosis and hemoglobin A1c (HbA1c) levels. 108 HCM patients were stratified by HbA1c levels into non-diabetic (n=38), prediabetic (n=40), and diabetic (n=30) subgroups, along with 30 healthy controls. All participants underwent 3.0-T CMR examination. Compared to non-diabetic HCM patients, prediabetic and diabetic HCM patients exhibited progressively higher mean T1 values and extracellular volume fractions (ECV) (*p* < 0.001). Similar trends were observed in hypertrophic myocardial regions, with diabetes patients showing pronounced fibrosis. Mean ECV exhibited a strong positive correlation with HbA1c levels (r = 0.634, *p* < 0.001). In the fully adjusted model, both T1 values and ECV demonstrated significant associations with HbA1c levels. Subclinical myocardial inflammation, as evidenced by elevated T1 and T2 values, was observed in prediabetic and diabetic patients but not in non-diabetic patients. Progression of myocardial fibrosis in HCM is linked to elevated HbA1c, especially in hypertrophied regions, even in prediabetic individuals. Subclinical myocardial inflammation was observed in HCM with glucose metabolism abnormalities. These findings underscore the importance of early glycemic control and the integration of CMR-based tissue characterization into HCM management strategies.

## Introduction

Hypertrophic cardiomyopathy (HCM) is the most common inherited primary cardiomyopathy, with a prevalence of approximately 0.2% in the general population (1, 2). The pathophysiological characteristic of HCM include myocardial fibrosis, myocardial hypertrophy, and cardiac dysfunction, which substantially increase the risks of heart failure, arrhythmias, and sudden cardiac death (3, 4).

Emerging evidence indicates that hyperglycemia may stimulate the proliferation of cardiac fibroblasts and the deposition of myocardial extracellular matrix *in vitro*, thereby inducing myocardial fibrosis (5). Clinical observations have demonstrated a graded increase in myocardial fibrosis severity across three distinct patient groups: non-diabetic, pre-diabetic, and diabetic individuals (6). Particularly in HCM, diabetes mellitus appears to exacerbate myocardial fibrosis, leading to further deterioration of cardiac function (7–11). However, the specific impact of varying degrees of glucose metabolism abnormalities on myocardial microstructure in HCM remains unclear. Additionally, it is uncertain whether subclinical myocardial changes are already present in prediabetic HCM patients and whether these changes worsen with the progression of glucose metabolism abnormalities.

Cardiac magnetic resonance (CMR) imaging is considered the gold standard for assessing cardiac structure and function (12). T1 mapping and extracellular volume fraction (ECV) evaluate diffuse myocardial fibrosis, while T2 mapping detects myocardial edema and inflammation (13, 14). Therefore, multiparametric-CMR, with its high sensitivity and specificity, is capable of identifying early changes in myocardial microstructure.

Given this background, the present study aims to systematically compare myocardial microstructural differences among non-diabetic, prediabetic, and diabetic HCM patients using multiparametric CMR. Furthermore, we explore the relationship between glycated hemoglobin A1c (HbA1c) levels and myocardial fibrosis.

## Methods

### Ethical approval

This study was approved by the Chengdu Third People’s Hospital Ethics Review Board (Ethics number: 2025-S-124). All procedures were performed in accordance with the principles of the Declaration of Helsinki. Due to its retrospective design, informed consent from participants was waived.

### Study population

**Figure 1:**
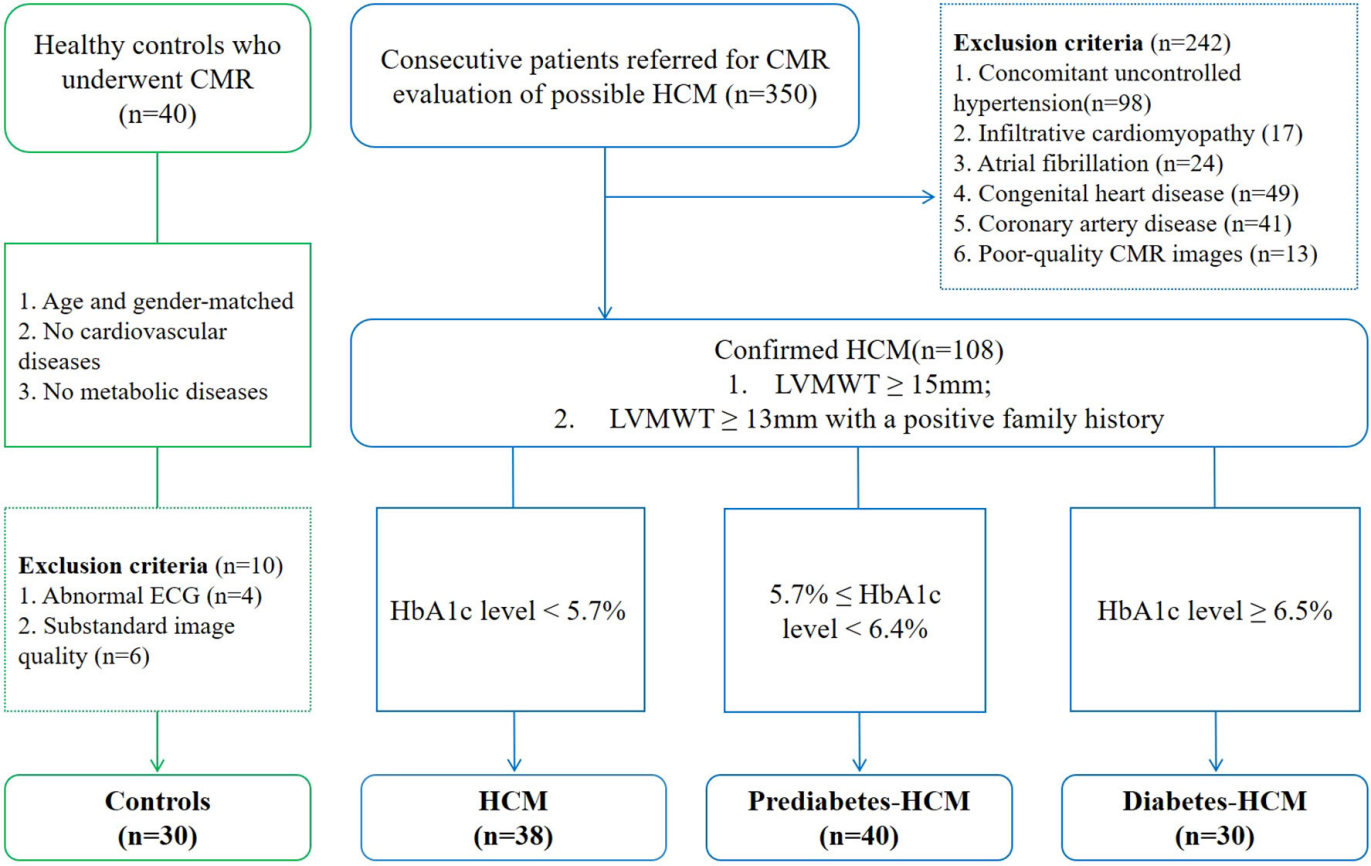
Subject flowchart. The flowchart shows the involvement of patients and controls. *CMR*, cardiac magnetic resonance; *ECG*, electrocardiogram; *HCM*, hypertrophic cardiomyopathy.

This retrospective study consecutively included 108 HCM patients who were evaluated with CMR imaging at Chengdu Third People’s Hospital from June 2020 to July 2024. Based on glucose metabolism status, the HCM patients were divided into three subgroups: Non-diabetic HCM group (n = 38): HbA1c level < 5.7%, prediabetic HCM group (n = 40): 5.7% ≤ HbA1c level < 6.4%, and diabetic HCM group (n = 30): HbA1c level ≥ 6.5% (15). Inclusion criteria for HCM diagnosis were defined as non-dilated left ventricular hypertrophy [left ventricular maximum wall thickness (LVMWT) ≥ 15 mm or LVMWT ≥ 13 mm with a positive family history] identified on CMR (16). Exclusion criteria included: (1) concomitant uncontrolled hypertension; (2) infiltrative cardiomyopathy; (3) persistent atrial fibrillation; (4) congenital heart diseases; (5) history of myocardial infarction or significant coronary artery stenosis (≥50%); (6) poor-quality CMR images or other factors interfering with measurements (**Figure 1**). A control group (n=30) was selected from a pre-existing database (17, 18), matched by age and sex to the HCM cohort. The control group served primarily as a reference for non-diabetic HCM patients, allowing us to identify myocardial tissue changes associated with HCM independent of metabolic abnormalities. The control group consisted of participants with no history of cardiovascular or metabolic diseases, as confirmed by clinical assessment and medical history.

### CMR imaging protocol

All participants underwent 3.0-T CMR (Magnetom Skyra, Siemens Healthcare, Erlangen, Germany) examination, following a standardized protocol that included cine imaging, pre- and post-contrast T1 mapping, T2 mapping, and late gadolinium enhancement (LGE) sequences. Cine imaging: electrocardiogram gated steady-state free precession sequences with three long-axis planes and sequential short-axis slices from the base to the apex of the left ventricle. And the typical imaging parameters were as follows: field of view (FOV) = 340×340 mm^2^; slice thickness = 6 mm; flip angle = 52°; time of repetition (TR) = 3.3 ms and time of echo (TE): 1.43 ms. T1 mapping: Modified Look-Locker inversion recovery (MOLLI) sequence was used to acquire short-axis slices at the basal, mid, and apical levels, with additional two- and four-chamber views for apical HCM patients. Images were obtained before and 10–20 minutes after intravenous administration of 0.15 mmol/kg gadolinium-based contrast (gadovist, Bayer Healthcare Pharmaceuticals). The MOLLI acquisition before contrast agent administration followed the 5(3)3 protocol during a breath hold. MOLLI images acquired after contrast agent administration followed the 4(1)3(1)2 protocol during a breath hold (19, 20). And the typical imaging parameters were as follows: FOV = 340×340 mm^2^; slice thickness = 8 mm; 7/8 phase partial Fourier acquisition; flip angle = 35°; TR = 354.77 ms and TE = 1.15 ms. T2 mapping: Balanced steady-state free precession (bSSFP) sequence with T2 preparation (21). And the following imaging parameters: FOV = 340×340 mm^2^; slice thickness = 8 mm; 6/8 phase partial Fourier acquisition; flip angle = 12°; TR = 242.95 ms and TE = 1.49 ms. LGE imaging: Phase-sensitive inversion recovery (PSIR) sequences were acquired 10 minutes after contrast injection to evaluate myocardial fibrosis (22). Imaging was performed in four-chamber, two-chamber, and short-axis views. The inversion time was individually adjusted to null the myocardium signal.

### CMR data analysis

Two independent radiologists (X.P. with >6 years of experience and J.G. with >15 years of experience) analyzed sequentially numbered CMR data in a blinded manner using Medis Suite MR software (QMass and QStrain, Leiden, The Netherlands).

The endocardial and epicardial borders of the left ventricle were manually delineated on cine images to quantify left ventricular mass, volume, and ejection fraction. LVMWT was defined as the greatest linear dimension at any site within the LV myocardium. Global longitudinal strain (GLS), global radial strain (GRS), and global circumferential strain (GCS) in LV were assessed using QStrain. Strain parameters were expressed as negative (shortening) or positive (thickening) values, reflecting deformation in longitudinal, radial, or circumferential directions.

**Figure 2:**
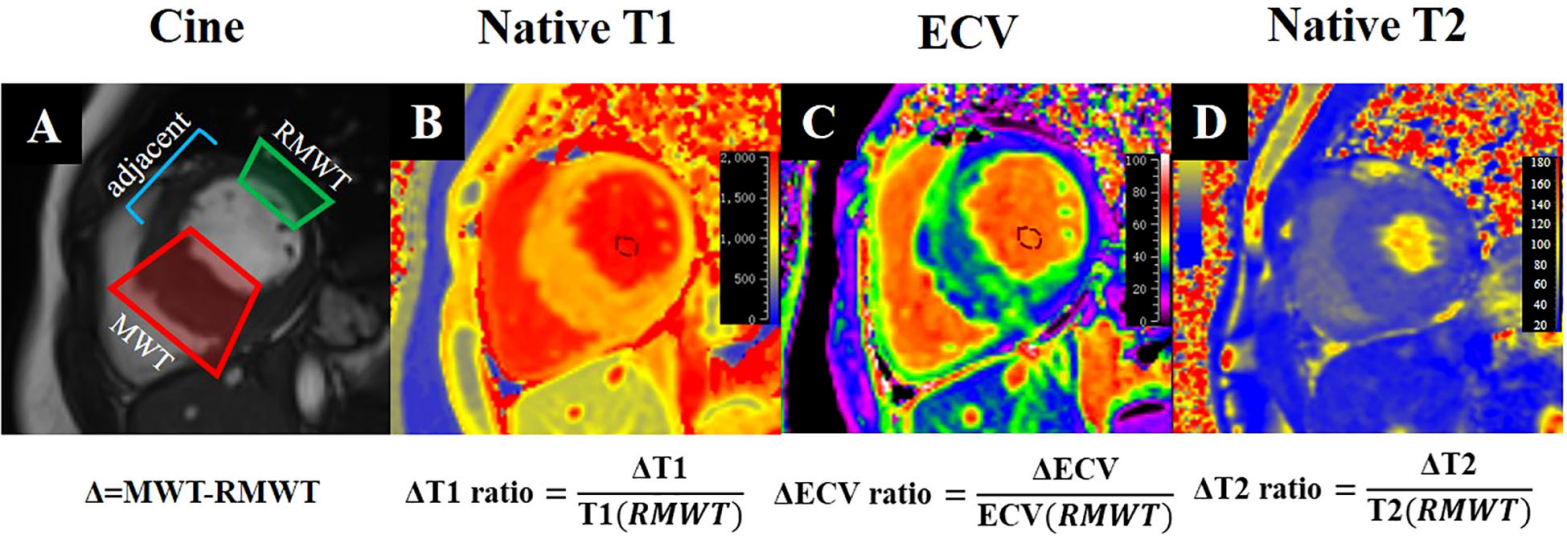
CMR tissue characterization in a 52-year-old diabetic male with interventricular septal HCM shows cine **(A)**, Native T1 **(B)**, ECV **(C)**, and Native T2 **(D)** imaging. The formulas beneath **(A)** define Δ as the difference between the MWT and RMWT regions. The formulas below **(B–D)** calculate the ΔT1, ΔECV, and ΔT2 ratios, enabling comprehensive quantitative assessment of myocardial tissue properties. *ECV*, extracellular volume; *MWT*, maximal wall thickness; *RMWT*, remote normal myocardial regions of MWT.

The LV myocardial T1 values and ECV were quantified using the American Heart Association 16-segment model, with measurements averaged from the basal, mid, and apical slices. Hematocrit level, obtained from venous blood samples collected within 24 hours of CMR examination, was used to calculate individual ECV (23). We further analyzed the T1 values and ECV of hypertrophic and remote myocardial regions. ΔT1 and ΔECV were calculated as the differences between the T1 and ECV values of hypertrophic and remote regions. The ΔT1 ratio was defined as the ratio of ΔT1 to the mean T1 values of remote normal regions, and the ΔECV ratio was defined as the ratio of ΔECV to the ECV of remote normal regions (**Figure 2**). And the definition of ΔT2 ratio follows a similar pattern. LGE was semiautomatically quantified by using the fullwidth half-maximum method with manual correction by using QMass (Medis Medical Imaging). Any obvious blood pool or pericardial partial volume artifacts were manually corrected.

### Statistical analysis

Continuous data were expressed as mean ± standard deviation (SD) or median (interquartile range [IQR]), while categorical data were presented as counts with corresponding percentages. Normality was assessed using the Shapiro-Wilk test and homogeneity of variances verified with Levene’s test. For normally distributed continuous variables, comparisons were conducted using one-way analysis of variance (ANOVA) followed by Tukey’s honestly significant difference (HSD) *post hoc* test, whereas the Kruskal–Wallis rank-sum test with Dunn-Bonferroni *post hoc* correction was employed for non-normally distributed variables, while categorical variables were compared using the Chi-square or Fisher’s exact test (when expected frequencies <5). The relationship between HbA1c levels and primary CMR findings was evaluated using Pearson or Spearman’s rank correlation methods. Multiple linear regression models were employed to estimate associations of CMR tissue characterization metrics with HbA1c levels. Multivariable adjustment was performed using a systematic covariate selection strategy incorporating three components: (1) clinically established demographic and metabolic confounders (age, sex, and body mass index (BMI)); (2) variables showing significant associations in univariate analyses (P<0.10), including hypertension; and (3) HCM-specific structural parameters (left ventricular mass index, maximal wall thickness) with established prognostic relevance in disease pathophysiology. Statistical analysis was performed by using SPSS software (IBM SPSS Statistics 25.0, Armonk, New York, USA) and GraphPad Prism (version 8.0; GraphPad Software, San Diego, California, USA). All tests were two-tailed test, and a *p* value <0.05 was used to determine statistical significance.

## Results

### General characteristics and cardiac functional parameters

The baseline characteristics of the participants are summarized in **Table 1**. There were no significant differences in age (*p*=0.416), sex (*p*=0.372), or BMI (*p*=0.07) among the four groups. Similarly, no significant differences were observed in conventional cardiovascular risk factors (smoking, drinking, hypertension, and dyslipidemia) across the HCM subgroups. Demographic and clinical information were collected from medical records. Most diabetic HCM patients (80%, 24 of 30) were treated with non-insulin medications, mainly metformin (43%, 13 of 30), and only 20% (6 of 30) of the patients were treated with insulin.

**Table 1:** Baseline clinical characteristics.

Variables	HCM (n=38)	Prediabetes-HCM (n=40)	Diabetes-HCM (n=30)	Healthy controls (n=30)	*P* value
Age (years)	59 ± 12	58 ± 10	60 ± 9	56 ± 9	0.416
Male, n(%)	27(71.1)	21(52.5)	17(56.6)	19(63.3)	0.372
BMI (kg/m^2^)	24.5 ± 3.5	25.4 ± 3.4	25.4 ± 3.5	23.4 ± 3.7	0.07
Heart rate (bpm)	68 ± 11	71 ± 14	73 ± 12	70 ± 11	0.456
Family history of HCM, n (%)	2(5.3)	1(2.5)	0(0.0)	-	0.631
Smoking, n(%)	17(44.7)	14(35.0)	11(36.6)	13(43.3)	0.789
Drinking, n(%)	13(34.2)	13(32.5)	8(26.6)	9(30.0)	0.918
Hypertension, n(%)	20(52.6)	21(52.5)	17(56.6)	0(0.0)*†‡	**<0.001**
Dyslipidemia, n(%)	10(26.3)	8(20.0)	8(26.6)	2(6.6)	0.168
HbA1c (%)	5.2 ± 0.3	6.0 ± 0.2*	7.7 ± 1.7*†	5.1 ± 0.4†‡	**<0.001**
Duration of diabetes	-	-	5.6 ± 5.8	-	**-**
TC (mmol/liter)	4.5 ± 1.1	4.2 ± 1.1	4.7 ± 2.2	4.5 ± 1.2	0.631
TG (mmol/liter)	2.0 ± 1.7	1.7 ± 1.1	1.7 ± 1.1	1.3 ± 0.6	0.157
LDL-C (mmol/liter)	2.6 ± 0.8	2.5 ± 0.9	2.5 ± 0.9	2.6 ± 0.7	0.901
HDL-C (mmol/liter)	1.3 ± 0.3	1.4 ± 0.4	1.3 ± 0.3	1.3 ± 0.4	0.373
Detectable hs-CRP (≥0.8 mg/L), n (%)	11(28.9)	17(42.5)	13(43.3)	10(33.3)	0.289
hs-CRP (mg/L), median (IQR)	1.9(1.2-4.1)	2.0(1.4-4.6)	4.2(2.3-7.2)	2.1(1.2-3.1)	0.126
hs-cTnT (ng/L)	16.6(8.2-23.3)	13.4(9.4-32.5)	16.2(10.8-41.7)	-	0.377
Medical therapy					
Beta-blockers, n(%)	29(76.3)	27(67.5)	24(80.0)	-	0.461
Calcium-channel blockers, n(%)	7(18.4)	10(25.0)	10(33.3)	-	0.370
ACEI/ARB, n(%)	14(36.8)	23(57.5)	17(56.7)	-	0.131
Statin, n(%)	25(65.8)	31(77.5)	21(70.0)	-	0.512
Metformin, n(%)	-	-	13(43.3)	-	-
SGLT2 inhibitor, n(%)	-	-	12(40.0)	-	-
Sulfonylurea, n(%)	-	-	4(13.3)	-	-
Acarbose, n(%)	-	-	6(20.0)	-	-
Insulin, n(%)	-	-	6(20.0)	-	-

Data are reported as mean ± SD or n (%) as appropriate.

Bold indicates *P* value <0.05. *P <0.05 vs. HCM; †*P <*0.05 vs. Prediabetes-HCM; ‡*P <*0.05 vs. Diabetes-HCM.

*BMI*, Body mass index; *TC*, Total Cholesterol; *TG*, Triglycerides; *LDL-C*, Low-Density Lipoprotein Cholesterol; *HDL-C*, High-Density Lipoprotein Cholesterol; *hs-CRP*, Hypersensitive C-reactive protein; *hs-cTnT*, high-sensitivity cardiac troponin T; *ACEI/ARB*, angiotensin-converting enzyme inhibitor or angiotensin receptor blocker; *SGLT2*, sodiumdependent glucose transporters 2.

The CMR findings are presented in **Table 2**. Compared to healthy controls, all HCM groups exhibited increased left ventricular mass index (LVMi), but no significant differences were found among the HCM subgroups. Diabetic HCM patients demonstrated significantly reduced left ventricular end-diastolic volume index (LVEDVi) and end-systolic volume index (LVESVi) compared to non-diabetic HCM patients. HCM patients exhibited significantly reduced LVGRS and LVGLS compared to healthy controls. Furthermore, as the severity of glucose metabolism abnormalities increased (from non-diabetic to prediabetic and diabetic states), there was a progressive decline in LVGRS (62.7 ± 7.5% vs. 50.2 ± 5.5% vs. 43.4 ± 6.2%, *p* < 0.001) and LVGLS (-23.2 ± 2.6% vs. -20.5 ± 3.6% vs. -18.1 ± 3.6%, *p* < 0.001) among HCM subgroups.

**Table 2:** CMR-Based cardiac function parameters.

Variables	HCM (n=38)	Prediabetes-HCM (n=40)	Diabetes-HCM (n=30)	Healthy Controls (n=30)	*P* value
LVMWT (mm)	17.7 ± 3.8	17.4 ± 3.3	17.2 ± 3.9	-	0.873
LVOT obstruction, n(%)	6(16)	14(35)	10(33)	-	0.121
LV mass (g)	118.6 ± 43.01	120.0 ± 47.4	110.98 ± 39.0	72.0 ± 15.3*†‡	**<0.001**
LV mass index (g/m^2^)	68.4 ± 24.3	70.7 ± 25.8	65.0 ± 20.3	41.8 ± 6.4*†‡	**<0.001**
LVEDVi (ml/m^2^)	68.2 ± 17.1	66.3 ± 15.4	57.3 ± 12.3*	66.6 ± 11.1	**0.014**
LVESVi (ml/m^2^)	25.0 ± 7.5	24.5 ± 9.5	19.7 ± 5.4*†	25.2 ± 5.1‡	**0.010**
LVSVi (ml/m^2^)	43.2 ± 10.9	42.0 ± 8.3	37.6 ± 8.8	41.4 ± 7.4	0.076
LVEF (%)	63.8 ± 5.5	63.9 ± 7.0	65.6 ± 5.9	62.2 ± 4.0‡	0.183
LVCOi l/(min*m^2^)	2.9 ± 0.8	2.9 ± 0.8	2.7 ± 0.7	2.9 ± 0.5	0.637
LVGRS (%)	62.7 ± 7.5	50.2 ± 5.5*	43.4 ± 6.2*†	80.1 ± 6.7*†‡	**<0.001**
LVGCS (%)	-23.4 ± 3.9	-23.1 ± 4.7	-24.5 ± 4.8	-24.2 ± 2.6	0.468
LVGLS (%)	-23.2 ± 2.6	-20.5 ± 3.6*	-18.1 ± 3.6*†	-26.6 ± 1.7*†‡	**<0.001**

Data are reported as mean ± SD or n (%) as appropriate.

Bold indicates *P* value <0.05. *P <0.05 vs. HCM; †*P <*0.05 vs. Prediabetes-HCM; ‡*P <*0.05 vs. Diabetes-HCM.

*CMR*, cardiac magnetic resonance*; LV*, left ventricular; *LVMWT*, LV maximal wall thickness; *LVOT*, LV outflow tract; *LVEDVi*, LV end-diastolic volume index; *LVESVi*, LV end-systolic volume index; *LVSVi*, LV stroke volume index; *LVEF*, LV ejection fraction; *LVCOi*, LV cardiac output index; *LVGRS*, LV global radial strain; *LVGCS*, LV global circumferential strain; *LVGLS*, LV global longitudinal strain.

### Myocardial tissue characterization via CMR

**Figure 3:**
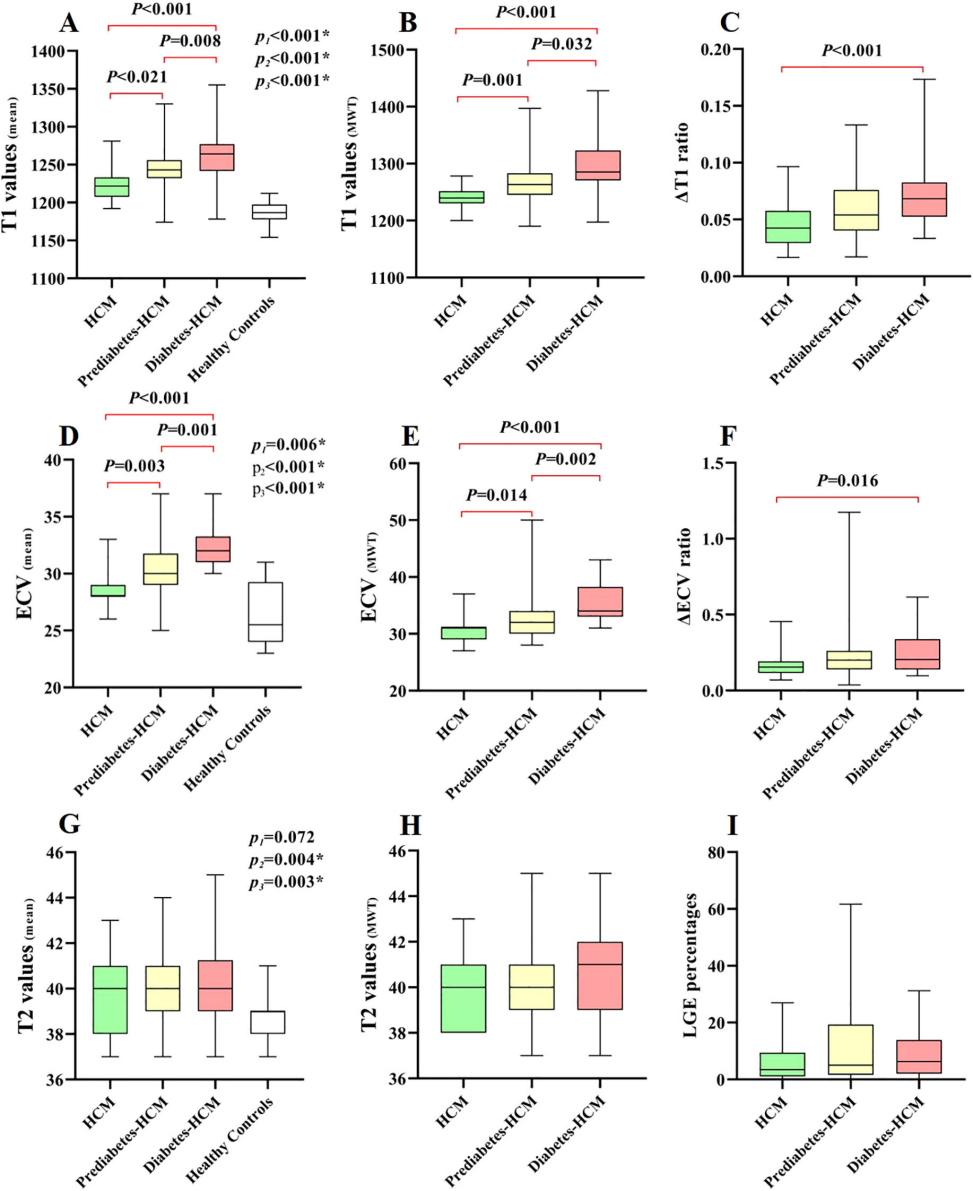
Box-whisker plots of different cardiac CMR parameters in HCM patients and healthy controls. P1, P2, and P3 denote comparisons between: 1) P1: HCM vs Healthy Controls; 2) P2: Prediabetes-HCM vs Healthy Controls; 3) P3: Diabetes-HCM vs Healthy Controls. The horizontal lines denote the 5th to 95th percentiles, the shaded boxes depict the first to third quartiles, and the central lines represent the median values. The plots demonstrate the differences in T1-related **(A–C)**, ECV-related **(D–F)**, T2-related **(G, H)** parameters, and LGE percentages **(I)** among different HCM subgroups and/or healthy controls. *LGE*, late gadolinium enhancement. * p value <0.05.

CMR tissue characterization results are shown in **Table 3**. The presence of LGE showed no significant differences among the HCM subgroups. The mean T1 values and ECV for HCM were greater than those of the healthy participants. Among the HCM subgroups, both mean T1 values and ECV increased progressively with worsening glycemic status (T1: 1223 ± 20 ms vs. 1241 ± 34 ms vs. 1263 ± 36 ms, *p* < 0.001; ECV: 28.5 ± 1.5% vs. 30.1 ± 2.4% vs. 32.1 ± 1.7%, *p* < 0.001), and a similar trend was observed in the hypertrophic myocardial regions. However, in the remote normal myocardial regions of hypertrophied segments, significant differences in T1 values and ECV were observed only between the diabetic and non-diabetic groups. Furthermore, compared to non-diabetic patients, the diabetic group demonstrated significantly higher ΔT1 ratio (0.72 ± 0.27 vs. 0.46 ± 0.22, *p* < 0.001) and ΔECV ratio (0.25 ± 0.14 vs. 0.16 ± 0.07, p < 0.001) in hypertrophic myocardial regions, while ΔT2 ratio showed no difference (**Figure 3**). The mean myocardial T2 values were significantly higher in prediabetic (40.0 ± 1.5 ms) and diabetic HCM patients (40.3 ± 1.8 ms) compared to healthy controls (38.9 ± 1.0 ms, *p* < 0.001), while non-diabetic HCM patients showed no significant difference.

**Table 3:** Myocardial tissue characterization with CMR.

Variables	HCM (n=38)	Prediabetes-HCM (n=40)	Diabetes-HCM (n=30)	Healthy Controls (n=30)	*P* value
Presence of LGE, n (%)	33(87)	34(85)	27(90)	-	0.822
LGE (%LV)	3.4(1.1-9.4)	5.0(1.5-18.3)	5.0(1.6-13.5)	-	0.420
LV blood T1 (ms)	1774 ± 134	1735 ± 174	1780 ± 150	1749 ± 117	0.291
RV blood T1 (ms)	1664 ± 116	1631 ± 167	1684 ± 146	1615 ± 122	0.197
T1 values-mean (ms)	1223 ± 20	1241 ± 34*	1263 ± 36*†	1187 ± 14*†‡	**<0.001**
T1 values-MWT (ms)	1240 ± 17	1268 ± 42*	1296 ± 47*†	1187 ± 14*†‡	**<0.001**
T1 values-RMWT (ms)	1186 ± 26	1197 ± 30	1210 ± 30*	1187 ± 14‡	**0.001**
ΔT1 ratio	0.463 ± 0.216	0.599 ± 0.262	0.717 ± 0.273*	-	**<0.001**
Hematocrit (%)	43.3 ± 5.0	42.2 ± 4.9	41.8 ± 5.4	42.9 ± 4.6	0.602
ECV-mean (%)	28.5 ± 1.5	30.1 ± 2.4*	32.1 ± 1.7*†	26.5 ± 2.7*†‡	**<0.001**
ECV-MWT (%)	30.7 ± 2.1	32.8 ± 3.9*	35.9 ± 3.5*†	26.5 ± 2.7*†‡	**<0.001**
ECV-RMWT (%)	26.3 ± 1.7	26.7 ± 2.1	28.8 ± 1.6*†	26.5 ± 2.7‡	**<0.001**
ΔECV ratio	0.164 ± 0.073	0.233 ± 0.191	0.248 ± 0.143*		**0.006**
T2 values-mean (ms)	39.6 ± 1.4	40.0 ± 1.5	40.3 ± 1.8	38.9 ± 1.0†‡	**0.002**
T2 values-MWT (ms)	39.8 ± 1.4	40.2 ± 1.6	40.6 ± 1.8	38.9 ± 1.0*†‡	**<0.001**
T2 values-RMWT (ms)	39.6 ± 1.3	39.9 ± 1.6	40.4 ± 1.5	38.9 ± 1.0†‡	**0.005**
ΔT2 ratio	0.006 ± 0.112	0.009 ± 0.121	0.125 ± 0.143	-	0.149

Data are reported as mean ± SD, median (IQR), or n (%) as appropriate.

Bold indicates *P* value <0.05. *P <0.05 vs. HCM; †*P <*0.05 vs. Prediabetes-HCM; ‡*P <*0.05 vs. Diabetes-HCM.

*LGE*, late gadolinium enhancement; *RV*, right ventricular; *MWT*, maximal wall thickness; *RMWT*, remote normal myocardial regions of MWT; *ECV*, extracellular matrix volume fraction.

### Correlation between myocardial fibrosis and HbA1c levels

**Figure 4:**
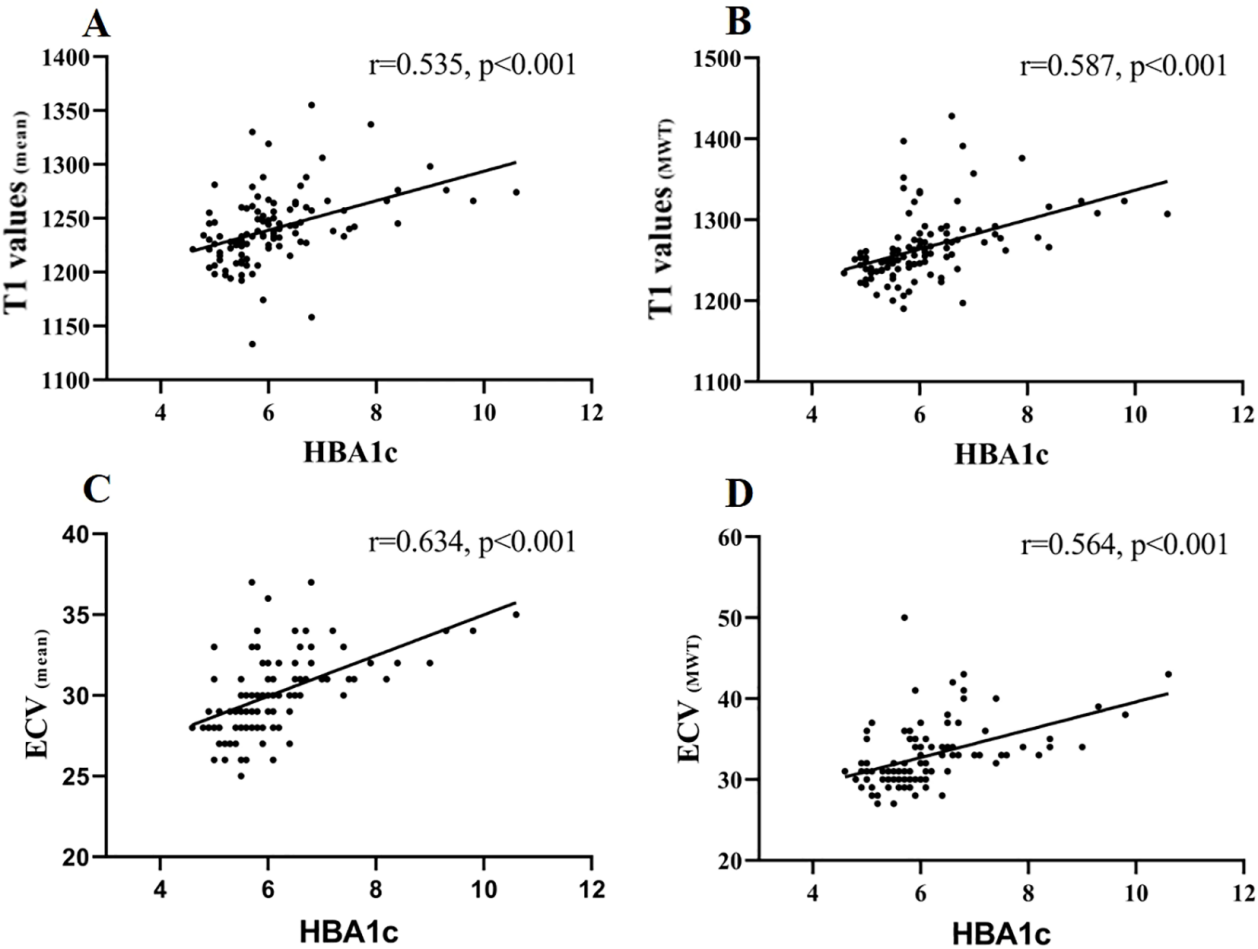
Correlation of T1 values (mean), T1 values (MWT), ECV (mean), and ECV (MWT) with HBA1c. Spearman’s correlations were **(A)** T1 values (mean) and HBA1c: r = 0.535, p < 0.001; **(B)** T1 values (MWT) and HBA1c: r = 0.587, p < 0.001; **(C)** ECV (mean) and HBA1c: r = 0.634, p < 0.001; **(D)** ECV (MWT) and HBA1c: r = 0.564, p < 0.001.

The correlation analysis demonstrated a strong positive association between mean ECV and HbA1c levels (r = 0.634, *p* < 0.001). Moderate correlations were also observed between HbA1c and mean T1 values, as well as T1 values and ECV at maximal wall thickness (r= 0.535, 0.587, and 0.564 respectively, all *p* < 0.001) (**Figure 4**), indicating that higher HbA1c is associated with greater myocardial fibrosis.

In the fully adjusted model (adjusted for age, sex, BMI, hypertension, left ventricular mass index and maximal wall thickness), both T1 values and ECV demonstrated persistent and significant associations with HbA1c levels (p < 0.001) (**Table 4**).

**Table 4:** Associations between CMR tissue characterization metrics and HBA1c.

CMR metrics	Model 1			Model 2			Model 3		
	*β (95% CI)*	Standardizes *β*	*P* value	*β (95% CI)*	Standardizes *β*	*P* value	*β (95% CI)*	Standardizes *β*	*P* value
T1 values (mean)	11.673(7.301-16.046)	0.460	**<0.001**	11.714(7.310-16.119)	0.462	**<0.001**	11.710(7.329-16.091)	0.461	**<0.001**
T1 values (MWT)	13.907(8.366-19.449)	0.440	**<0.001**	13.957(8.374-19.540)	0.441	**<0.001**	14.187(8.625-19.750)	0.448	**<0.001**
ECV values (mean)	0.860(0.553-1.167)	0.482	**<0.001**	0.882(0.578-1.186)	0.494	**<0.001**	0.887(0.581-1.193)	0.497	**<0.001**
ECV (MWT)	1.100(0.586-1.613)	0.389	**<0.001**	1.117(0.602-1.632)	0.395	**<0.001**	1.092(0.582-1.602)	0.386	**<0.001**
T2 values (mean)	0.052(-0.178-0.281)	0.044	0.655	0.049(-0.182-0.280)	0.042	0.673	0.078(-0.154-0.309)	0.066	0.506
T2 values (MWT)	0.128(-0.106-0.362)	0.106	0.280	0.124(-0.112-0.359)	0.103	0.299	0.147(-0.089-0.384)	0.122	0.220

Results are the association of CMR tissue characterization metrics with HBA1c from linear regression model, expressed as their corresponding 95% CIs, *P* values, and standardized beta coefficients. Model 1: age, male, and BMI; Model 2: Model 1+ hypertension; Model 3: Model 2+ LVMWT, and LV mass index.

## Discussion

By investigating the role of CMR characterization in assessing the impact of glycemic states on myocardial microstructure in HCM patients, we demonstrated that: (a) A progressive increase in left ventricular myocardial fibrosis, as assessed by T1 values and ECV, with worsening glucose metabolism abnormalities from non-diabetic to prediabetic and diabetic states. Notably, fibrotic changes in the remote regions of hypertrophied myocardium exhibited significant differences solely between diabetic and non-diabetic patients. In addition, diabetic HCM patients showed a greater fibrosis burden in hypertrophic myocardial regions compared to remote regions; (b) Subclinical myocardial inflammation, indicated by elevated T1 and T2 values, was observed in prediabetes-HCM and diabetes-HCM patients but not in non-diabetic HCM patients; (c) Glycemic abnormalities are associated with further deterioration of myocardial deformation.

### Myocardial fibrosis and its correlation with HbA1c levels

Myocardial fibrosis is a hallmark pathological feature in HCM, contributing to adverse cardiovascular event (24–27). Previous research evidence has established a potential dose-response relationship between myocardial fibrosis and plasma glucose levels (6), while compelling clinical data further demonstrate that diabetes mellitus significantly exacerbates myocardial fibrosis progression in HCM patients (10, 11). Our findings corroborate previous observations, revealing a graded progression of myocardial fibrosis, as evidenced by elevated T1 values and ECV, across the spectrum from non-diabetic to prediabetic and diabetic HCM patients. Even after adjusting for multiple potential confounders, HbA1c levels remain an independent determinant of myocardial fibrosis. However, the absence of significant differences in LGE among HCM subgroups, suggests that the observed fibrotic changes might represent subtle and diffuse expansion of the extracellular matrix at an early stage, rather than irreversible myocardial focal scarring detectable by LGE (28).

Interestingly, the study appears to indicate that, compared to the non-diabetic group, diabetic group exhibited significantly elevated fibrosis not only in hypertrophied myocardial segments but also in remote regions, whereas the prediabetic group showed no notable difference in fibrosis in remote areas compared to the non-diabetic group. Furthermore, the substantial elevation of ΔT1 and ΔECV ratios in diabetic HCM patients highlights that hypertrophied myocardial regions bear a disproportionately higher burden of fibrosis. This indicates that diabetes not only amplifies fibrosis in regions already burdened by hypertrophy but also drives diffuse myocardial remodeling throughout the left ventricle in HCM. The underlying mechanisms may be attributed to hyperglycemia, which induces diffuse myocardial fibrosis in HCM patients through multiple pathways, including oxidative stress, pro-inflammatory state, growth factor secretion, neurohumoral activation, deposition of advanced glycation end-products, and activation of the renin-angiotensin-aldosterone system (29). Hyperglycemia increases reactive oxygen species, activating profibrotic signals like TGF-β and promoting fibroblast activity. AGEs accumulation further stimulates inflammation and fibrosis. RAAS activation contributes to vasoconstriction and myocardial remodeling. Together, these processes drive diffuse fibrosis, especially in hypertrophied myocardium with microvascular dysfunction (30). Additionally, hypertrophied myocardial regions in HCM exhibit more severe microvascular dysfunction and low-grade inflammation compared to remote regions (31). Hyperglycemia exacerbates endothelial dysfunction in the microvasculature (10), leading to impaired vasodilation and further reduction in blood perfusion, which consequently aggravates fibrosis in the hypertrophied myocardial regions.

The observed positive correlation between the severity of myocardial fibrosis, as measured by T1 values and ECV, and HbA1c levels suggests that the prediabetic stage may accelerate the progression of myocardial fibrosis in HCM patients. This association may be attributed to hyperglycemia-induced proliferation of cardiac fibroblasts and an increase in extracellular matrix production (5, 32).

### Subclinical myocardial inflammation and its clinical implications

In a study of 674 HCM patients, Xu et al. demonstrated significantly elevated T2 values (33), potentially attributed to myocardial inflammatory cell infiltration and microvascular dysfunction (34, 35). In contrast, we did not observe a significant increase in T2 values in our cohort of non-diabetic HCM patients. This discrepancy may be explained by the relatively small sample size of 38 cases in our study, which could reduce statistical power and increase the likelihood of random variability and uncertainty.

However, our study revealed that prediabetic and diabetic HCM patients exhibited subclinical myocardial inflammation, as evidenced by significantly elevated T1 and T2 values compared to healthy controls. Furthermore, diabetes itself has been shown to activate myocardial inflammatory processes through mechanisms such as hyperglycemia-induced oxidative stress and cytokine release (36, 37), and the coexistence of diabetes and HCM may synergistically exacerbate myocardial inflammatory responses, potentially due to the combined effects of microvascular dysfunction in HCM and the pro-inflammatory environment induced by hyperglycemia.

Based on our findings of the additive effects of HCM and hyperglycemia on myocardial inflammation, we hypothesize that glucose metabolism abnormalities exacerbate myocardial inflammatory cell infiltration in HCM patients, leading to myocardial inflammation. This hypothesis is supported by the elevated T1 and T2 values observed in our cohort, which are indicative of tissue inflammation. However, this mechanism requires further validation through histological studies or myocardial biopsy.

Although CRP and hs-cTnT levels did not differ significantly across groups, differences in CMR parameters were observed, suggesting that CMR may be more sensitive than conventional biomarkers in detecting subtle myocardial changes associated with glycemic dysregulation at an early stage.

### Impaired myocardial strain with glycemic abnormalities

Hajdu et al. (38) reported that in patients with type 1 diabetes mellitus, current HbA1c levels remained an independent predictor of impaired GLS and GCS, even after adjustment for age and hypertension. Similarly, other studies have demonstrated that diabetic patients exhibit early subclinical myocardial dysfunction, often reflected in reduced strain values, even in the absence of overt structural heart disease (39–41). Studies have shown that both HCM and diabetic patients can experience varying degrees of impaired myocardial deformation. In line with these findings, our results demonstrated a progressive decline in GRS and GLS from non-diabetic to prediabetic and diabetic HCM patients, revealing subclinical left ventricular dysfunction. The observation that strain impairment is already evident in prediabetic HCM patients further supports the hypothesis that metabolic dysregulation, particularly glucose abnormalities, plays a critical role in exacerbating myocardial dysfunction in HCM. This decline is likely associated with increased myocardial fibrosis and inflammation (42, 43), as reported in both diabetic cardiomyopathy and advanced HCM phenotypes.

### Clinical implications

This study highlights the clinical importance of glycemic assessment in HCM patients. The association between worsening glycemic status and myocardial remodeling suggests that even prediabetes may contribute to subclinical dysfunction. Early metabolic screening and multiparametric CMR can aid in risk stratification and guide timely interventions, such as lifestyle or metabolic therapy, to slow disease progression and improve outcomes.

### Limitations

Our study has several limitations. First, as a retrospective single-center study with a relatively modest sample size, our analysis may have been underpowered to detect clinically relevant but subtle differences, particularly in subgroup comparisons. The inherent constraints of retrospective designs - including potential selection bias (e.g., possible overrepresentation of more severe cases at our tertiary referral center), unmeasured confounders (such as temporal variations in clinical management protocols), and incomplete medication adherence data - limit causal inference. Although no significant LGE differences were observed among glycemic subgroups, this negative finding may reflect limited statistical power for subgroup analyses rather than true biological equivalence, especially given the potential influence of glucose-lowering agents (e.g., GLP-1 receptor agonists or SGLT2 inhibitors) on myocardial remodeling that was not fully accounted for. Second, the presence of subclinical myocardial inflammation was inferred from T1 and T2 mapping rather than being confirmed by myocardial biopsy. Third, we only performed CMR assessments at a single time point, precluding the evaluation of dynamic changes in myocardial microstructure. Therefore, the findings should be interpreted with caution. Future studies should address these limitations by enrolling larger, multicenter cohorts with adequate statistical power, incorporating longitudinal imaging, and specifically evaluating the impact of glycemic abnormalities and metabolic therapies on myocardial tissue remodeling in HCM patients.

### Conclusion

In summary, this study underscores the critical impact of glycemic abnormalities on myocardial microstructure in HCM patients, as revealed by tissue-characteristic CMR. Myocardial fibrosis progression is strongly associated with elevated HbA1c levels, particularly in hypertrophied regions, even in prediabetic individuals. Additionally, subclinical myocardial inflammation was evident in prediabetic and diabetic HCM patients. Our findings emphasize the role of early glycemic control in mitigating myocardial fibrosis and support the use of CMR-based tissue characterization in HCM management.

## Data Availability

The raw data supporting the conclusions of this article will be made available by the authors, without undue reservation.
